# Disrupted Eye Gaze Perception as a Biobehavioral Marker of Social Dysfunction: An RDoC Investigation

**Published:** 2020-09-10

**Authors:** Ivy F. Tso, Carly A. Lasagna, Kate D. Fitzgerald, Costanza Colombi, Chandra Sripada, Scott J. Peltier, Timothy D. Johnson, Katharine N. Thakkar

**Affiliations:** 1 Department of Psychiatry, University of Michigan, Ann Arbor, Michigan, MI 48109, USA; 2 Department of Psychology, University of Michigan, Ann Arbor, Michigan, MI 48109, USA; 3 Functional MRI Laboratory, University of Michigan, Ann Arbor, Michigan, MI 48109, USA; 4 Department of Biomedical Engineering, University of Michigan, Ann Arbor, Michigan, MI 48109, USA; 5 Department of Biostatistics, University of Michigan, Ann Arbor, Michigan, MI 48109, USA; 6 Department of Psychology, Michigan State University, East Lansing, Michigan, MI 48824, USA

**Keywords:** social cognition, social functioning, gaze perception, functional magnetic resonance imaging, psychosis, autism-spectrum disorders, social anxiety

## Abstract

Social dysfunction is an intractable problem in a wide spectrum of psychiatric illnesses, undermining patients’ capacities for employment, independent living, and maintaining meaningful relationships. Identifying common markers of social impairment across disorders and understanding their mechanisms are prerequisites to developing targeted neurobiological treatments that can be applied productively across diagnoses and illness stages to improve functional outcome. This project focuses on eye gaze perception, the ability to accurately and efficiently discriminate others’ gaze direction, as a potential biomarker of social functioning that cuts across psychiatric diagnoses. This premise builds on both the monkey and human literatures showing gaze perception as a basic building block supporting higher-level social communication and social development, and reports of abnormal gaze perception in multiple psychiatric conditions accompanied by prominent social dysfunction (e.g., psychosis-spectrum disorders, autism-spectrum disorders, social phobia). A large sample (*n* = 225) of adolescent and young adult (age 14–30) psychiatric patients (regardless of diagnosis) with various degrees of impaired social functioning, and demographically-matched healthy controls (*n* = 75) will be recruited for this study. Participant’s psychiatric phenotypes, cognition, social cognition, and community functioning will be dimensionally characterized. Eye gaze perception will be assessed using a psychophysical task, and two metrics (precision, self-referential bias) that respectively tap into gaze perception disturbances at the visual perceptual and interpretation levels, independent of general deficits, will be derived using hierarchical Bayesian modeling. A subset of the participants (150 psychiatric patients, 75 controls) will additionally undergo multimodal fMRI to determine the functional and structural brain network features of altered gaze perception. The specific aims of this project are three-fold: (1) Determine the generality of gaze perception disturbances in psychiatric patients with prominent social dysfunction; (2) Map behavioral indices of gaze perception disturbances to dimensions of psychiatric phenotypes and core functional domains; and (3) Identify the neural correlates of altered gaze perception in psychiatric patients with social dysfunction. Successfully completing these specific aims will identify the specific basic deficits, clinical profile, and underlying neural circuits associated with social dysfunction that can be used to guide targeted, personalized treatments, thus advancing NIMH’s Strategic Objective 1 (describe neural circuits associated with mental illnesses and map the connectomes for mental illnesses) and Objective 3 (develop new treatments based on discoveries in neuroscience and behavioral science).

## BACKGROUND

### Social Dysfunction Is an Intractable Problem across Mental Illnesses

Mental illness is the leading cause of disability in the US [1]. A key factor contributing to disability in mental illness is deficits in social functioning, which significantly undermine patients’ ability to obtain/maintain employment and meaningful relationships, two key components of recovery. Thus, improving social functioning among individuals with mental disorders has significant potential to reduce the burden of mental illness. However, existing treatments fall far short of meeting this need. Medications, the predominant treatment for most mental illnesses in the US, fail to improve social functioning. Psychotherapies, while generally more effective at improving social functioning, are insufficient for addressing the problem due to difficulties with access and adapting to different disorders, the substantial time required, and their tremendous variation in efficacy across individuals. To develop treatments that can be applied efficiently across diagnoses and individuals, improving the outcome of mental illnesses, we first need to identify key markers of social functioning across disorders and to understand their underlying mechanisms. Previous work has indicated that eye gaze perception, the ability to discriminate others’ gaze direction, is one such potential biomarker of social functioning that spans multiple diagnoses.

### Disturbed Gaze Perception as a Hypothesized Mechanism of Social Dysfunction across Diagnoses

The gaze of others is a ubiquitous social cue that conveys information about the gazer’s attention and intention. Thus, the ability to *accurately* and *efficiently* discriminate others’ gaze direction is critical to understanding others and the complex social world [2]. Altered gaze perception can disrupt higher-level social cognitive abilities, causing difficulties in social functioning [3]. Multiple studies, including our own, have shown that gaze perception is disrupted in schizophrenia [4–7] and is associated with deficits in higher-order social cognition [6,7]. Abnormal gaze perception has also been reported in other psychiatric disorders that are accompanied by social dysfunction, including bipolar disorder [7], autism-spectrum disorders (ASD; [8,9]), and social phobia [10–14]. Disturbed gaze perception is not an all-or-nothing phenomenon that appears only in above-threshold psychopathologies. Rather, as suggested by findings of subclinical studies, the degree of gaze perception abnormalities is proportional to the levels of psychosis proneness [15], autism traits [16], and social anxiety [12]. Together with the fact that these disorders (and their subclinical features) are highly comorbid [17–19], prevalent among both psychiatric patients and the general population [20–22], and all accompanied by social dysfunction, it is plausible that altered eye gaze perception represents a common pathway to social dysfunction across individuals regardless of diagnoses. Since most previous work has focused on chronic patient samples, showing abnormal gaze perception early in the course of mental illness would strengthen the hypothesis that abnormal gaze perception is a cause rather than a consequence of social dysfunction.

### Understanding the Cognitive and Neural Underpinnings of Abnormal Gaze Perception Would Form Novel Treatment Targets to Improve Social Functioning

Gaze perception involves not only visual processing, but also higher-level cognition such as attentional control and self-referential processing. Disruption to any of these could result in atypical gaze perception. For example, disrupted early visual processing could lead to noisy (i.e., imprecise) gaze perception. Alternatively, one can have precise perception but a bias to perceive gaze as self-directed when it is not. Previous gaze perception studies in psychiatric populations typically focused on accuracy against a presumed “ground truth” and rarely explicitly dissociated the cognitive components involved (e.g., precision, bias) from one another [8]. Dissociating these cognitive processes can help us understand the similarities and differences between psychopathologies, as well as identifying the sources of individual differences. To achieve this, we designed a task using a psychophysics method to systematically study gaze perception as a function of gaze direction [6]. This task allows us to derive a psychometric function to characterize each individual’s gaze perception, and then use the slope and position of this function to index precision and bias respectively. Using this method, we demonstrated that abnormal gaze perception in schizophrenia is characterized by reduced precision as well as a stronger self-referential bias compared to controls [6,7]. These disturbances showed differential symptom/functional correlates [6,23], suggesting that they measure independent constructs and pick up different deficits. It remains to be determined if precision and bias are similarly altered in other disorders exhibiting abnormal gaze perception, since existing studies typically used methods that are descriptive (e.g., lower accuracy, wider “cone of gaze”) and do not disentangle the cognitive processes involved in gaze perception [9–12,15]. It is likely that altered gaze perception in different psychopathologies arises from differential levels of visual and self-referential processing deficits. This claim is based on previous findings that show early visual processing to be noisy in psychosis [24,25], abnormally precise in ASD [26] and normal in social anxiety, whereas self-referential processing is shown to be hyperactive in psychosis [27] and social anxiety [28,29] but reduced or absent in ASD [30]. Since psychosis proneness, autism traits, and social anxiety span the full range of normal-abnormal human behavior, considering them as three dimensions (instead of discrete categories) of psychopathology and investigating how they map onto specific gaze perception disturbances would help identify mechanisms common and unique to these psychopathologies and advance our understanding and prediction of individual differences. Such approach would enable the examination of possible interactive or moderating effects between these psychopathology dimensions on different aspects of gaze perception.

Neuroimaging studies in healthy individuals indicate that gaze perception is subserved by complex interactions between the visual system and other brain networks, including the fronto-parietal control and salience networks [31,32]. Few studies of gaze perception in psychiatric populations have specifically investigated neural correlates [33–37]. However, neuroimaging studies of social and emotion processing in disorders displaying abnormal gaze perception and social dysfunction (e.g., psychosis, ASD, and social anxiety) have revealed commonalities of abnormal functions in brain regions (e.g., medial frontal cortex [MFC], superior temporal sulcus [STS], inferior parietal lobule [IPL], amygdala, and anterior insula; [27,28,35,38]) that are also implicated in gaze perception. This suggests that abnormalities in gaze perception and other socioemotional functions have overlapping neural origins. This underscores the value of better understanding the neural underpinnings of altered gaze perception in psychiatric patients with social dysfunction, as the knowledge would help identify specific neural circuits as treatment targets for improving social functioning. As evidence increasingly suggests that abnormal social behavior is a result of altered network organizations and connections [39–44], rather than localized brain dysfunctions, a complete account of disturbed gaze perception will need to encompass both intra- and inter-network dynamics.

Our pilot work using the analytic technique of dynamic causal modeling (DCM) [45] of fMRI data provides some preliminary understanding of the brain dynamics involved in gaze perception disturbances in schizophrenia. We found defective bottom-up processes (weakened response to sensory input in the visual cortex, and reduced feedforward connectivity within the visual areas) during face processing, as well as abnormally increased MFC inhibition of the visual cortex when processing gaze specifically. We interpret the latter finding as a mechanism to compensate for impaired data-driven perception by increasing reliance on higher-level cognition to determine the self-referential nature of gaze. These findings are consistent with the observation in healthy individuals that experimentally introduced visual impediments not only make gaze perception less precise, but also more self-biased [46], likely because higher-level cognition (a prior expectation that others’ gaze is directed to self [47]) is engaged as a compensation mechanism [48]. Together, these findings provide a framework for understanding the relationships between specific cognitive processes and abnormal gaze perception, and suggest that the visual system (and perhaps the prefrontal cortex) should be engaged as treatment targets to improve social functioning in schizophrenia. More work is needed to determine whether the same mechanisms are involved in other mental illnesses with social deficits.

## SIGNIFICANCE

This project will investigate altered gaze perception as a key biobehavioral marker of social dysfunction. We will adopt a trans-diagnostic approach and study psychiatric patients with varying degree of impaired social functioning regardless of diagnosis. By focusing on the 14–30 age group where most mental illnesses emerge and escalate, we will be able to determine the presence and extent of gaze perception abnormalities near illness onset and how they change as a function of illness stage. We will use psychophysics methods to quantify precision and self-referential bias of gaze perception. Given previous reports of abnormal visual scanning patterns during face viewing in schizophrenia [49], autism [50], and social anxiety [51], we will use eye tracking to rule out the possibility that altered gaze perception is due to reduced fixation on the eye region. We will map gaze perception disturbances onto three dimensions of psychopathology (psychosis proneness, autism traits, and social anxiety), which are prevalent in psychiatric patients and bear significant impact on social functioning. We will use multimodal fMRI to identify brain network abnormalities underlying gaze perception disturbances. The findings of this project will advance NIMH’s Strategic Objective 1 (describe neural circuits associated with mental illnesses and map the connectomes for mental illnesses) and Objective 3 (develop new treatments based on discoveries in neuroscience and behavioral science), by fulfilling the specific aims of the grant.

## SPECIFIC AIMS

### Aim 1: Determine the Generality of Gaze Perception Disturbances in Psychiatric Patients with Prominent Social Dysfunction

#### Hypothesis 1a:

Psychiatric patients will show reduced precision and increased self-referential bias as compared with controls, as shown previously in schizophrenia.

#### Hypothesis 1b:

Disrupted gaze perception in psychiatric patients is not due to abnormal visual scanning.

### Aim 2: Map Behavioral Indices of Gaze Perception Disturbances to *Dimensions* of Psychiatric Phenotypes and Core Functional Domains

#### Hypothesis 2a:

Precision of gaze perception is negatively associated with psychosis proneness and positively with autism traits, and is correlated with basic visual functions (gain control, visual integration).

#### Hypothesis 2b:

Self-referential bias during gaze perception is positively associated with social anxiety and psychosis proneness and negatively with autism traits.

#### Hypothesis 2c:

Gaze perception precision and self-referential bias independently contribute to social cognition and community function in psychiatric patients.

### Aim 3: Identify the Neural Correlates of Altered Gaze Perception in Psychiatric Patients with Social Dysfunction Using fMRI

#### Hypothesis 3a:

Psychiatric patients will exhibit abnormal visual cortical function and prefrontal-visual connectivity, as previously observed in schizophrenia.

#### Hypothesis 3b:

Among patients, visual cortical dysfunction is associated with reduced precision of gaze perception, and abnormal prefrontal-visual connectivity with self-referential bias.

## METHODS

### Overview

We will recruit 225 psychiatric patients having various degrees of social dysfunction (including but not limited to patients with psychosis-spectrum disorders, autism-spectrum disorders, and social anxiety) and 75 age- and sex-matched controls. Sample sizes were determined using power analyses based on prior work and after accounting for attrition (see below for details). We will recruit participants between the ages of 14–30, where most mental illnesses emerge and escalate. All participants will complete a comprehensive battery to characterize psychiatric phenotypes, cognition, social cognition, and community functioning. Eye gaze perception will be assessed using a psychophysical task, and two metrics indexing independent cognitive processes involved in gaze perception (precision, self-referential bias) will be derived using hierarchical Bayesian modeling (HBM). A subset of the participants (150 psychiatric patients, 75 controls) will additionally undergo multimodal fMRI to determine the brain network features of altered gaze perception. Only a subset of patients will complete fMRI because, based on our experience, a significant portion (25–40%) of patient participants would not meet fMRI criteria due to issues such as weight, medical conditions, inability to tolerate scanner noise or enclosed space, metal in body, etc. These patients are often otherwise eligible and should be included for the behavioral part of the study (Aims 1 & 2) to enhance the representativeness of the sample and the generalizability of the results.

### Participants and Recruitment

All participants will be aged 14–30 recruited from hospital clinics, existing research registries, social media campaigns, and internet/community advertisement. Psychiatric patients will have a psychiatric condition for which they seek help in a primary/mental health care setting and at least moderate difficulty in social functioning, determined by: sum of ≥4 on the Work and Social Adjustment Scale (WSAS) social items AND score of ≤6 on the Global Functioning Scales social sub-scale [52]. Controls will not exhibit any social impairment (WSAS-social < 4 AND global functioning-social > 6), take psychotropic medication, or have any history of DSM-5 diagnosis.

To address the issue of neurodevelopment, we will evenly sample the entire age range (i.e., 1/3 from ages 14–17, 1/3 from ages 18–25, and 1/3 from ages 26–30). This will allow a sufficient number of participants across the developing and developed ranges, enabling statistical analyses to model the linear and quadratic effects of age in behavioral and brain function. Then, in order to ensure the sample will be rich in the three psychopathology dimensions of interest (psychosis-proneness, autism traits, social anxiety), we will require 60% of the patient sample meet criteria for a schizophrenia-spectrum disorder, ASD, or social phobia (with at least 20% for each).

### Assessments

The assessment battery comprehensively characterizes participants’ psychiatric phenotypes, general intellectual functioning, social functioning, social cognition, basic aspects of visual perception, and gaze perception using validated instruments suitable for individuals aged 14 and above.

#### Psychiatric phenotypes

We will assess major psychiatric diagnoses and measure psychopathology trait dimensions we hypothesize to be related to gaze perception. Diagnoses will be determined using the Mini-International Neuropsychiatric Interview (MINI for DSM-5) or the MINI Kid and Kid Parent version for participants < 18. The three psychopathology dimensions of interest (psychosis proneness, autism traits, and social anxiety) will be measured using: the Peters Delusion Inventory [53], Cardiff Anomalous Perception Scale [54], and the Scale for the Assessment of Negative Symptoms (SANS;[55]) for psychosis proneness; the Autism Spectrum Quotient (ASQ) [56] and the Autism Diagnostic Observation Schedule Second Edition (ADOS-2) [57] for autism traits; and the Social Phobia Inventory (SPIN) [58,59] and Social Anxiety Disorder Dimensional Scale (SAD-D)[60] for social phobia.

#### General intellectual functioning

The two-subset form (Vocabulary, Matrix Reasoning) of the Wechsler Abbreviated Scale of Intelligence Second Edition (WASI-II) [61] will be used.

#### Social functioning

Assessment will be conducted using self-report, clinician-rated, and informant-rated measures: Social Adjustment Scale—Self-Report: Short (SAS-SR: Short) [62], Global Functioning: Social and Role [63], and Social Responsiveness Scale (SRS)[64].

#### Social cognition

Low-level (perceptual) to higher-level (empathy, social inference) social cognition will be assessed using: Reading the Mind in the Eyes test [65], Empathy Quotient (EQ) [66], and Awareness of Social Inference Test (TASIT) [67].

#### Visual perception

We will probe two facets of basic visual perception (contrast sensitivity, visual integration) that, when disrupted, have been associated with aberrant gaze perception [23,46]. Contrast sensitivity refers to the ability to distinguish an object from its background; it will be assessed using the SLOAN low contrast charts. Visual integration refers to the process linking the individual local elements to form a global, holistic representation, underpinning Gestalt perception that is critical to object and facial recognition [24]. Visual integration will be assessed using the Jitter Orientation Visual Integration Task (JOVI) [68].

#### Gaze perception

**Figure 1.**
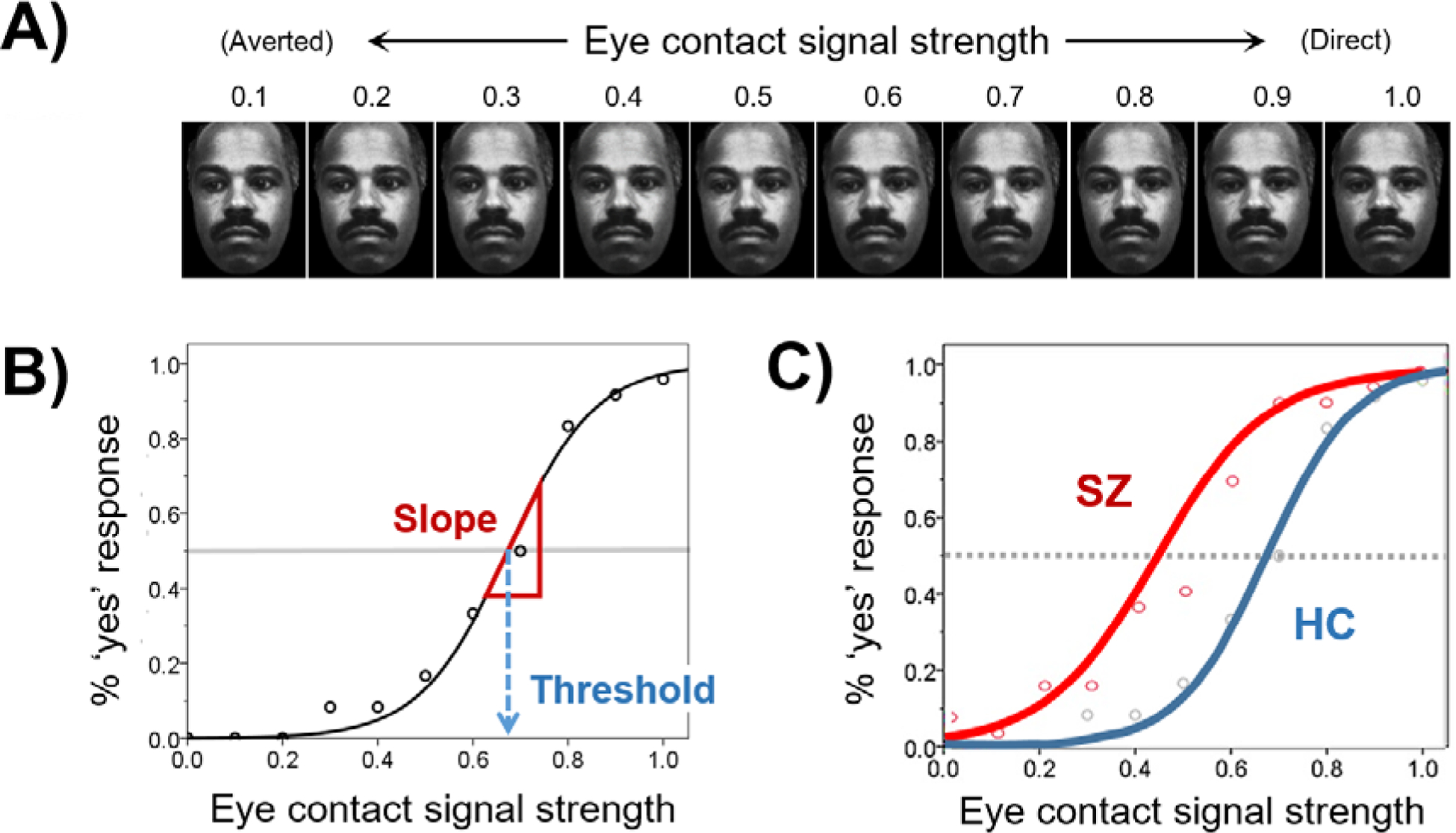
The psychophysical eye gaze perception (GAZE) task. SZ = schizophrenia; HC = healthy controls. (**A**) Sample GAZE task stimuli. (**B**) Response at individual level is fitted with a logistic function to derive threshold (self-referential tendency) and slope (perceptual precision). (**C**) *Prototypical* gaze perception curve for schizophrenia and controls based on results obtained from our previous studies [6,7] showing reduced threshold (increased self-referential bias) and slope (reduced precision) in SZ relative to HC.

We will use a psychophysical eye gaze perception task (GAZE) similar to the ones used in our previous studies, in which individuals with a primary psychotic disorder generally exhibited decreased perceptual precision and increased self-referential bias relative to controls (e.g., [6,7]; see Figure 1C). This task will present naturalistic face images of several actors with forward and deviated head orientations, depicting a range of incremental gaze angles (0°–12°) from eyes looking directly at the viewer to eyes averted away from the viewer in left/right directions (Figure 1A). Head orientation will also be manipulated, in addition to gaze angle, because it is known to impact gaze perception [69–71] and data suggest that forward/deviated faces capture differential aspects of gaze processing [72]. In the task, participants will see the faces one at a time in a pseudo-randomized order. To each face, they indicate whether they feel the face is looking at them or not, according to their first impression. Each face appears for 2 s, separated by an inter-trial fixation cross.

During GAZE and TASIT, eye movements will also be recorded using a video-based eye tracker capable of tracking up to 2000 Hz.

### fMRI Acquisition Protocol

Scanning will take place on a 3.0 T GE Discovery MR750 scanner (Milwaukee, WI) with a 32-channel receiver array Nova Medical head coil (Wilmington, MA). Total time in the scanner, including task and scan setup, is approximately 75 min.

#### Structural scans

For each subject, a whole-brain T1-weighted structural image (3D SPGR sequence, 1mm isotropic resolution, FOV = 25.6, TI = 1060, TE = Min Full, FA = 8°, 320 × 320 matrix, parallel acceleration factor = 2) and a T2-weighted structural image (Cube sequence, 1 mm isotropic resolution, TR = 4100, TE = 60, 256 × 256 matrix) will be acquired.

#### Diffusion-weighted imaging

DWI data will be acquired according to the NIMH Adolescent Brain Cognitive Development ABCD protocol (multiband factor = 3, 102 directions [*b* = 0, 5 volumes; *b* = 500, 6 directions; *b* = 1000, 15 directions; *b* = 2000, 15 directions; *b* = 3000, 60 directions] 1.7 mm resolution, 81 slices, FOV = 24, TR = 7400, TE = Min, 140 × 140 matrix).

#### Functional scans

**Figure 2.**
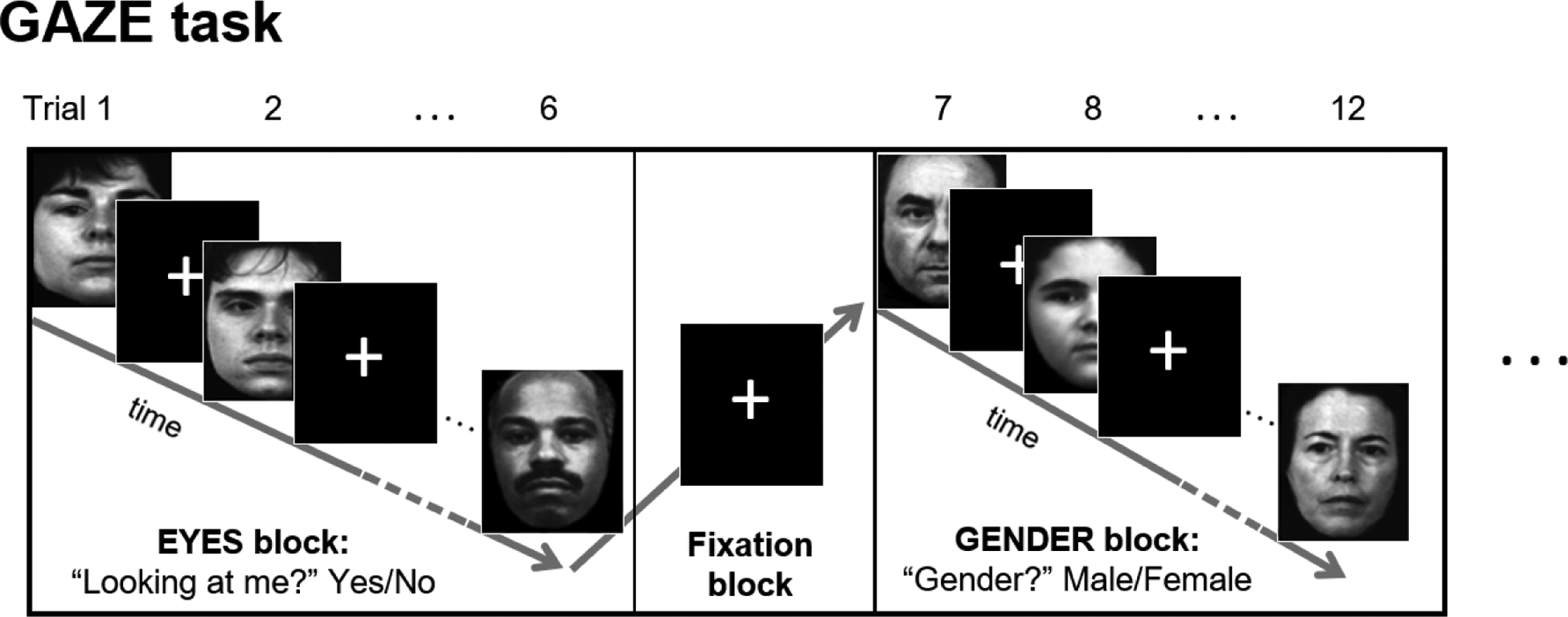
The fMRI version of the Eye Gaze Perception Task (GAZE) in a mixed (blocked event-related) design. EYES and GENDER blocks (19.8–24.4 s) alternate with a fixation block in between. During EYES blocks, participants are presented with face stimuli and must indicate whether the actor is ‘looking at me’ (yes/no). During GENDER blocks, participants are presented with the same stimuli, but, instead, must indicate the gender of the actor (male/female). Stimuli are face images depicting a range of gaze angles from looking directly at the viewer, to looking away from the viewer, in precise increments. Within each block, gaze angle and gender are randomized; each face is presented for 1.5 s and separated from the next face by a random jitter (1.6–3.9 s).

These will be acquired using a multi-band slice accelerated (factor = 6) gradient echo sequence (2.4 mm isotropic resolution, FOV = 23, TR = 800, TE = 30; FA = 52˚, 96 × 96 matrix). Spin echo-based b0 fieldmaps (TR = 7400, TE = 80) with forward and reverse phase encoding will be acquired before each of the functional task scan (GAZE) and a 10-minute resting-state scan to correct spatial distortion in the images. The GAZE task used during fMRI scanning will be a blocked event-related adaptation of the behavioral GAZE task (Figure 2).

### fMRI Data Processing Protocol

Imaging data (task, rest, and DTI) will be processed in accordance with the Human Connectome Project (HCP) preprocessing pipelines [73]. For task functional data, strict quality control procedures are in place to evaluate movement, excluding runs where the standard deviation of realignment parameters (summed across three rotational axes and three translational directions) is >0.5 mm translation and >0.2 degrees rotation. At the first-level analysis, motion parameters will be entered as nuisance regressors. For resting-state functional connectivity data, time-series will be band-pass filtered (0.01–0.10 Hz), high motion frames censored, and physiological noise removed using a PCA-based noise correction method (CompCor) [74]. DTI data will be manually inspected for significant artifacts as well as undergo an automated quality assessment protocol based on temporal signal-to-noise ratio, mean/maximum voxel intensity outlier count, and mean motion [75]. Diffusion-weighted scans will be pre-processed and analyzed using FSL 5.0 (FMRIB’s Software Library, www.fmrib.ox.ac.uk/fsl). Scans will be realigned to the b0 image using affine registration and eddy current correction will be applied. DTI analyses must be performed in native space as diffusion gradients are specified in this space; however, regions of interest (ROIs) are created and group analyses will be performed in standard (MNI) space. In order to transform ROIs into each subject’s native space, the T1-weighted structural volume will be realigned to the mean b0-weighted image and subsequently normalized to MNI space. The inverse warping parameters from this step will be used to transform ROIs from MNI space to native space.

### Statistical Analysis

Our overall analysis plan consists of three main parts corresponding to the three specific aims of the grant.

#### Aim 1: Determine the generality of gaze perception disturbances in psychiatric patients with social dysfunction

##### Gaze perception metrics.

Two aspects of gaze perception (slope—indexing precision; threshold—indexing self-referential bias) will be derived from the behavioral responses on GAZE and compared between patients and controls using HBM. Responses on the task are either yes or no, so the number of “yes” responses (endorsement of eye contact), Y, for each gaze angle will be modeled as a random variable that follows a binomial probability distribution dependent on θ (an unknown value between 0 and 1 underlying the probability of Y) and *N* (the number of completed trials):

$${\text{Y}}_{i,j,k,m}\sim \text{Binomial}\left(\theta_{i,j,k,m},N_{i,j,k,m}\right)$$

where *i* indexes the participant, *j* is the gaze angle (of all gaze angles used), *k* is the head orientation (1 = forward, 2 = averted), and *m* is the group (1 = control, 2 = patient). As demonstrated in our prior work, the probability of Y varies with gaze angle and approximates a logistic function. Since Y increases as the gaze angle approaches 0° (direct gaze), gaze angles will be converted to “eye-contact signal strength,” *X*, on a 0–1 scale where 0 corresponds to the averted-most gaze angle and 1 to gaze angle of 0° (direct gaze). Then, θ is linked to *X* via a logit link function using two parameters, α and β, given by Equation 1:

(1) $$\text{logit}\left(\theta_{ij,k,m}\right)=\alpha_{i,k,m}+\beta_{i,k,m}\cdot X_{j,m}$$

Parameters α and β are then modeled to come from a normal distribution centered around the mean of the participant’s corresponding group m for each head orientation *k*:

$$\alpha_{i,k,m}\sim \text{Normal}\left[{\left(\mu_{\alpha }\right)}_{k,m},{\left(\tau_{\alpha }\right)}_{k,m}\right]$$

$$\beta_{i,k,m}\sim \text{ Normal }\left[{\left(\mu_{\beta }\right)}_{k,m},{\left(\tau_{\beta }\right)}_{k,m}\right]$$

where μ and τ denotes, respectively, the mean and precision (reciprocal of variance) of the normal distribution. The posteriors of the mean parameters (α’s, β’s, and μ’s) are then estimated using improper and uninformative priors, and precision parameters (τ’s) using gamma priors. Next, the two gaze perception metrics, self-referential bias and precision, will be respectively estimated by calculating the threshold and slope of the logistic function when “yes” response is given 50% of the time (Figure 1B). Markov Chain Monte Carlo (MCMC) simulation will be performed to sample the posterior distribution of the parameters and estimates of interest. One-tailed group (HC − Patient > 0) and sex (F − M > 0) differences with posterior probability >95% will be considered credible. We expect patients will show reduced precision and increased self-referential bias compared with controls (Hypothesis 1a).

##### Visual scanning patterns during GAZE.

To determine the effect of visual scanning on gaze perception, eye-tracking data will be analyzed. Using separate HBMs, the number and duration of fixations on the eye region (represented with Poisson and gamma distributions, respectively) of the face stimuli during GAZE will be compared between the patient and control groups. We hypothesize that gaze perception disturbances in patients cannot be accounted for by abnormal visual scanning (Hypothesis 1b), thus expecting no group differences between number and duration of fixation in the eye region. If group differences in visual scanning exist, then we will re-estimate the group differences in gaze perception metrics by adding visual scanning variables as fixed effect terms to Equation (1) of the HBM. We expect that after accounting for the individual differences in visual scanning, patients will still show reduced precision and increased self-referential bias compared with controls.

#### Aim 2: Map behavioral indices of gaze perception disturbances to dimensions of psychiatric phenotypes and core functional domains

To obtain more reliable measures of the psychopathology dimensions, we will use principal component analysis (PCA) to extract a common latent variable underlying data generated using different scales or from different sources for each psychopathology dimension. Individual estimates of gaze perception precision obtained in Aim 1 will be correlated with the psychosis proneness and autism traits factors, as well as basic visual perception measures. We hypothesize that gaze perception precision will be negatively associated with psychosis proneness and positively with autism traits, and correlated with basic visual perception functions (Hypothesis 2a). Individual estimate of self-referential bias during gaze perception will be correlated positively with social anxiety and psychosis proneness and negatively with autism traits (Hypothesis 2b). Additionally, we will also explore the relationship between the three psychopathology dimensions and gaze perception metrics via model comparison; specifically, we will include the three psychopathology factor scores in the HBM in Aim 1 and compare model fit (deviance information criterion, DIC) of the full and reduced models (i.e., with and without the psychopathology dimensions, respectively). Finally, we will use PCA to extract a common component underlying the three social functioning measures. This social functioning factor will then serve as the dependent variable of a hierarchical regression model, into which the two gaze perception metrics will be entered as predictors one at a time. We expect that gaze perception precision and self-referential bias will account for unique variance in social functioning (Hypothesis 2c).

#### Aim 3: Identify neural correlates of altered gaze perception in psychiatric patients with social dysfunction

Analyses of the neuroimaging data will consist of two parts. The first part will examine effective connectivity, resting state functional connectivity (rsfc), and structural connectivity separately. The second part will use a multivariate method to build predictive models integrating brain connectivity data across modalities.

##### Effective connectivity.

DCM will be used to uncover brain dynamics during gaze processing. We will consider a biologically plausible network consisting of 4 volumes of interest (VOIs) informed by our preliminary findings—secondary visual cortex (V2), posterior superior temporal sulcus (pSTS), inferior parietal lobule (IPL), and medial frontal cortex (MFC), which are self-connected and connected intrinsically to one another bi-directionally, and V2 will be modeled to receive driving visual input. We will examine how the intrinsic connections and their modulation by gaze discrimination differ between patients and controls. Analyses will be conducted using the HCP CIFTI file format and the associated grayordinates spatial coordinate system [73]. Time-series will be extracted from the 4 VOIs, identified using the Gaze-Gender contrast, and then entered into SPM12 for DCM analyses. Bayesian model averaging (BMA) [76] will be performed to robustly estimate parameters by weighting each possible model’s posterior probability. We hypothesize reduced driving input into V2, weakened feedforward connections from V2, and aberrant gaze modulatory effect on the MFC-V2 feedback connection in patients compared with controls. These 3 parameter estimates will be compared across groups using independent-sample *t*-tests, corrected for 3 comparisons. These parameters will then be correlated with the 2 gaze perception metrics, to test the hypothesis that reductions in driving input and feedforward connections from V2 will be correlated with reduced gaze perception precision, while aberrant gaze modulation on top-down connections from MFC will be correlated with more self-biased gaze perception.

##### Resting-state functional connectivity.

Cortical surface and subcortical regions, in grayordinates space [73], will be parcellated based on the Gordon et al. [77] and Seitzman et al. [78] parcellation schemes, and assigned to 14 networks highly resembling the Power networks [79]. Pearson’s product-moment correlation coefficients will then be calculated pairwise between time courses for each of the parcels, producing connectivity matrices for all subjects. Fisher’s r-to-z transformation will be applied. Group comparisons will focus on intra-network connectivity within the Visual Network (VisN) and inter-network connectivity between the Vis, cingulo-opercular, dorsal attention, and salience networks. We expect patients to show reduced intra-VisN connectivity, associated with reduced precision in gaze perception, and aberrant inter-network connectivity, associated with self-referential bias.

##### Structural connectivity.

We will use probabilistic tractography [80,81] to assess anatomical pathways between V2, pSTS, IPL, and MFC—identified using the method described in the DCM analyses above and converted from surface space (CIFTI) to volume space (NIFTI). Probabilistic tractography will be run between the ROIs within the visual system (V2, pSTS, IPL) and between these visual ROIs and the frontal ROI (MFC) following methods used in previous studies [82,83]. Single-subject results for each of the probabilistic tracts will be transformed to MNI space in order to perform group analyses. For each subject, mean fractional anisotropy, mean diffusivity, axial diffusivity, and radial diffusivity within each of the group-thresholded tracts will be extracted. These measures are thought to reflect different aspects of the biological microstructure [84]. We will compare these measures between the patient and control groups, and examine their correlations with the 2 gaze perception metrics and 3 psychopathology dimensions.

##### Multimodal predictive modeling.

We will apply the Brain Basis Set (BBS) method [85,86] to identify components integrating brain information across modalities that best predict gaze perception performance and social functioning. Effective connectivity, resting-state functional connectivity, and DTI measures will be input as predicting variables to identify components that best predict the gaze perception metrics (slope, threshold) and social functioning. A 10-fold cross-validation will be used.

### Power Analysis

#### Aim 1

Our prior work showed that the posterior distributions of group differences between controls and schizophrenia patients in the gaze perception measures (threshold, slope) were normally distributed. This characteristic allows us to use the posterior mean and variance to infer the population parameters. We base our sample size calculation on the posterior estimates of threshold because they generally had smaller effect sizes than those of slope, thus ensuring the sample size estimate to be large enough to detect group differences in both measures. The posterior mean and variance for the control—schizophrenia group difference in threshold obtained from a sample of 101 was 0.097 and 0.001849, respectively; therefore, the population mean and variance are estimated to be 0.097 and 0.1867. Since the psychiatric patients to be studied in this project will overall be less severely ill than schizophrenia patients and come from a wider illness severity spectrum, we conservatively assume the population mean of control–patient difference to be smaller (×0.5) and variance larger (×2). Given these assumptions, the total sample size required to detect one-tailed group difference (control > patient) with 95% credibility is *N* = 262. Allowing a conservative attrition or unusable data rate of 13%, we will recruit 300 participants (225 patients and 75 controls, at a 3:1 patient-to-control ratio). This patient size of 225 is larger than the minimum number (*N* > 193) required to detect sex difference within the patient group with 95% credibility, based on our posterior estimates of sex difference in schizophrenia (mean = 0.188, variance = 0.01924, *N* = 47) and assumptions of half of the effect size, two times the variance in this study, and 10% attrition or unusable data.

#### Aim 2

Our prior work showed that correlations between gaze perception measures and social functioning in schizophrenia patients and healthy controls ranged from *ρ* = 0.21 to 0.71. A sample size of 225 patients (or 300 total participants when analyzed as a normal-abnormal continuum) is sufficient to detect correlations as small as *ρ* = 0.24 (or *ρ* = 0.20) with 80% power at alpha <0.05, assuming 10% attribution/unusable data rate and Bonferroni correction for 10 multiple comparisons.

#### Aim 3

In DCM model selection Bayesian approaches as applied in the effective connectivity analyses are used to compute one’s confidence in the presence of an effect. Therefore, power analyses have less meaning in the context of the Bayesian inversion schemes. However, subsequent hypothesis testing regarding group comparison of model parameters is based on classical frequentist inference. Our pilot work suggested group differences of Cohen’s *d* > 0.45 in the connectivity parameters pertinent to the DCM hypotheses in the proposed project. A sample of 150 patients and 75 controls is sufficient to detect group differences with 80% power at alpha < 0.05, Bonferroni corrected for 3 multiple comparisons, and with 10% unusable data. Here, we use a 2:1 patient-to-control ratio to strike a balance between efficiency (requiring fewer total subjects than a 3:1 ratio to detect group differences) and having a large enough patient sample to detect clinical and functional correlations. This sample size is sufficient to detect group differences in rsfc and DTI analyses of medium effect sizes, which are the typical magnitudes reported in large studies or meta-analyses of clinical and subclinical psychosis and ASD [39,87,88].

## DISCUSSION & FUTURE DIRECTIONS

### Patient Heterogeneity

The patient sample in this study is by design heterogeneous as we are seeking to investigate a phenomenon across diagnostic boundaries. The diversity in psychiatric symptoms, illness chronicity, severity, and comorbidity allow us to map how behavioral and neural correlates of gaze perception vary as a function of these important variables. However, such heterogeneity could also dilute group differences between patients and controls. For the gaze perception metrics, if the group differences do not reach the arbitrary level of 95% credibility, the posterior probability estimates of such group differences yielded by HBM would still be informative. If the posterior probabilities indicate ambiguous evidence for group differences (<75%), we will conduct additional analyses to investigate if only a subset of patients, for example, those with specific diagnoses (e.g., schizophrenia-spectrum disorders) or high on specific psychopathology dimensions (e.g., psychosis proneness), show clear behavioral deficits in gaze perception compared with controls. Additionally, rather than dividing the participants into patients vs. controls, we can treat them as individuals along multiple psychopathology-normal continuums and analyze the data accordingly. We can similarly apply these two alternative strategies to the fMRI data. Therefore, whether our hypotheses of group differences are supported or not, the results of this project will inform the characteristics of patients who exhibit gaze perception disturbances and would likely benefit from intervention targeting gaze perception deficits.

### Effects of Brain Development

The brain experiences significant developmental changes until age 25. Inclusion of participants aged 14–25 raises the question about confounds due to brain development, which needs to be specifically addressed. Besides matching the patients and controls for age and sex, we will make special effort to recruit approximately one-third of each group from ages 14–17, one-third from ages 18–25, and one-third from ages 26–30, so that we will have a sufficient number of participants across the developing/developed ranges to enable analyses that include the modeling of linear and quadratic effects of age in behavioral and brain function. We will model behavioral and neural growth curves in across development and will collect information on pubertal stage to enable analyses examining the effect of pubertal hormones.

### Medication Confounds

Many patients will be on one or more psychotropic medications. Although psychotropic medications on the market generally have limited effect on social cognition, we will take steps to minimize potential medication confounds on the results. We will partially address this problem by: (1) comparing behavioral and brain measures of gaze perception between patients taking and not taking different types of medication (e.g., antidepressants, mood stabilizers, antipsychotics) as well as medicated vs. unmedicated patients; and (2) correlating behavioral and brain measures of gaze perception with medication load index [89] and normalized antipsychotics dosage [90]. Since medication regimens will vary according to the individual’s symptom profile, this may confound the analyses mapping different gaze perception disturbances onto psychopathology dimensions. Setting the upper age limit of participants at 30 will help minimize systematic differences between diagnostic groups due to different medication regimens (e.g., higher antipsychotic use in patients with psychotic symptoms), which tend to increase with duration of illness. If gaze perception disturbances are present in recent-onset patients and to the similar degree as in patients with longer duration of illness, at least we can rule out the possibility of long-term medications causing gaze perception abnormalities.

### Future Directions

Findings from the proposed project will set the stage for further mechanistic and intervention research in gaze perception and social cognition in psychopathologies.

#### Computational models

Individual differences in perception are a result of individual differences in both prior expectations and sensory functioning. Gaze perception can be readily understood in this Bayesian framework, and a computational approach can be used to derive the specific abnormalities contributing to the observed disturbances in gaze perception associated with specific dimensions of psychopathology. This proposed project will facilitate this future direction by providing empirical data to estimate linear scaling factors required in the Bayesian computational models, for example, the constants that link performance on the visual perception tasks to the parameters of the likelihood function or psychopathology measures to the prior function in the computational model.

#### Experiments to confirm causality

The neuroimaging findings of this proposed project will reveal the contribution of specific neural circuits to abnormal gaze perception. To establish the causal role of these brain regions/connectivity in abnormal gaze perception, we can employ TMS in future work to elicit a temporary, virtual lesion in one of these regions (e.g., MFC) in healthy individuals and assess the changes in behavior and brain dynamics. If the results mimic those observed in patients with altered gaze perception (e.g., self-biased gaze perception, abnormal modulation MFC of the visual cortex), this would provide strong, convergent evidence that justifies treatment trials targeting such brain region(s).

#### Targeted treatments

This project will uncover the cognitive and neural characteristics related to abnormal gaze perception in psychiatric patients displaying social dysfunction, setting a stage for the development of targeted, personalized interventions to improve social cognition and functioning. Interventions will be in the form of adaptive cognitive training (e.g., targeting visual processing and/or self-referential processing), combined with brain stimulation (e.g., TMS, HD-tDCS) to enhance and accelerate treatment effects. For example, if the person shows universally weakened visual cortical response, then an intervention should focus on training paradigms and brain stimulation designed to improve bottom-up processes. If abnormal gaze perception appears to stem from abnormal self-referential processing, then an intervention should focus on paradigms (e.g., theory of mind tasks) and brain stimulation sites (e.g., MFC) that strengthen top-down processes. If both bottom-up and top-down deficits are indicated, then an intervention should target both. The findings of the multimodal brain connectivity prediction model may be used to match individual patients to treatment (training paradigms/brain stimulation site) and dose.

### Impact

Successfully completing the specific aims of the grant will identify the specific basic deficits, clinical profile, and underlying neural circuits associated with social dysfunction. These will guide new treatments combining specific cognitive training (visual and/or self-referential processing) and brain stimulation (e.g., TMS, HD-tDCS) to improve social functioning in patients, achieving NIMH’s goal of improving the outcome of mental illnesses.

## References

[1] Murray CJL. The State of US Health, 1990–2010: Burden of Diseases, Injuries, and Risk Factors. JAMA. 2013;310(6):591–608. 23842577 10.1001/jama.2013.13805 PMC5436627

[2] Emery NJ. The eyes have it: the neuroethology, function and evolution of social gaze. Neurosci Biobehav Rev. 2000;24(6):581–604. 10940436 10.1016/s0149-7634(00)00025-7

[3] Campbell R, Lawrence K, Mandy W, Mitra C, Jeyakuma L, Skuse D. Meanings in motion and faces: Developmental associations between the processing of intention from geometrical animations and gaze detection accuracy. Dev Psychopathol. 2006;18(1):99–118. 16478554 10.1017/S0954579406060068

[4] Rosse R, Kendrick K, Wyatt R. Gaze discrimination in patients with schizophrenia: preliminary report. Am J Psychiatry. 1994;151(6):919–21. 8185005 10.1176/ajp.151.6.919

[5] Hooker C, Park S. You must be looking at me: The nature of gaze perception in schizophrenia patients. Cogn Neuropsychiatry. 2005;10(5):327–45. 16571465 10.1080/13546800444000083

[6] Tso IF, Mui ML, Taylor SF, Deldin PJ. Eye-contact perception in schizophrenia: Relationship with symptoms and socioemotional functioning. J Abnorm Psychol. 2012;121(3):616–27. 22250658 10.1037/a0026596

[7] Yao B, Mueller SA, Grove TB, McLaughlin M, Thakkar K, Ellingrod V, Eye gaze perception in bipolar disorder: Self-referential bias but intact perceptual sensitivity. Bipolar Disord. 2018;20(1):60–9. 29168603 10.1111/bdi.12564 PMC5807101

[8] Pantelis PC, Kennedy DP. Deconstructing atypical eye gaze perception in autism spectrum disorder. Sci Rep. 2017;7(1):14990. 29118362 10.1038/s41598-017-14919-3 PMC5678184

[9] Forgeot D’Arc B, Delorme R, Zalla T, Lefebvre A, Amsellem F, Moukawane S, Gaze direction detection in autism spectrum disorder. Autism. 2017;21(1):100–7. 27132008 10.1177/1362361316630880

[10] Chen T, Nummenmaa L, Hietanen JK. Eye Contact Judgment Is Influenced by Perceivers’ Social Anxiety But Not by Their Affective State. Front Psychol. 2017;8:373. 28344569 10.3389/fpsyg.2017.00373 PMC5344928

[11] Gamer M, Hecht H, Seipp N, Hiller W. Who is looking at me? The cone of gaze widens in social phobia. Cogn Emot. 2011;25(4):756–64. 21547777 10.1080/02699931.2010.503117

[12] Jun YY, Mareschal I, Clifford CWG, Dadds MR. Cone of direct gaze as a marker of social anxiety in males. Psychiatry Res. 2013;210(1):193–8. 23769393 10.1016/j.psychres.2013.05.020

[13] Schulze L, Renneberg B, Lobmaier JS. Gaze perception in social anxiety and social anxiety disorder. Front Hum Neurosci. 2013 Dec 16;7:872. 24379776 10.3389/fnhum.2013.00872 PMC3863960

[14] Schulze L, Lobmaier JS, Arnold M, Renneberg B. All eyes on me?! Social anxiety and self-directed perception of eye gaze. Cogn Emot. 2013;27(7):1305–13.. 23438447 10.1080/02699931.2013.773881

[15] Wastler HM, Lenzenweger MF. Cone of gaze in positive schizotypy: Relationship to referential thinking and social functioning. Personal Disord. 2018;9(4):324–32. 28627901 10.1037/per0000258

[16] Madipakkam AR, Rothkirch M, Dziobek I, Sterzer P. Access to awareness of direct gaze is related to autistic traits. Psychol Med. 2019;49(6):980–6. 29947310 10.1017/S0033291718001630

[17] Bejerot S, Eriksson JM, Mörtberg E. Social anxiety in adult autism spectrum disorder. Psychiatry Res. 2014;220(1–2):705–7. 25200187 10.1016/j.psychres.2014.08.030

[18] McEnery C, Lim MH, Tremain H, Knowles A, Alvarez-Jimenez M. Prevalence rate of social anxiety disorder in individuals with a psychotic disorder: A systematic review and meta-analysis. Schizophr Res. 2019;208:25–33. 30722947 10.1016/j.schres.2019.01.045

[19] Kincaid DL, Doris M, Shannon C, Mulholland C. What is the prevalence of autism spectrum disorder and ASD traits in psychosis? A systematic review. Psychiatry Res. 2017;250:99–105. 28152400 10.1016/j.psychres.2017.01.017

[20] Kelleher I, Cannon M. Psychotic-like experiences in the general population: characterizing a high-risk group for psychosis. Psychol Med. 2010;41(1):1–6. 20624328 10.1017/S0033291710001005

[21] Fehm L, Beesdo K, Jacobi F, Fiedler A. Social anxiety disorder above and below the diagnostic threshold: prevalence, comorbidity and impairment in the general population. Soc Psychiatry Psychiatr Epidemiol. 2007;43(4):257–65. 18084686 10.1007/s00127-007-0299-4

[22] Constantino JN, Todd RD. Autistic Traits in the General Population. Arch Gen Psychiatry. 2003;60(5):524. 12742874 10.1001/archpsyc.60.5.524

[23] Tso IF, Carp J, Taylor SF, Deldin PJ. Role of visual integration in gaze perception and emotional intelligence in schizophrenia. Schizophr Bull. 2014;40(3):617–25. 23666503 10.1093/schbul/sbt058 PMC3984511

[24] Butler PD, Silverstein SM, Dakin SC. Visual Perception and Its Impairment in Schizophrenia. Biol Psychiatry. 2008;64(1):40–7. 18549875 10.1016/j.biopsych.2008.03.023 PMC2435292

[25] Keane BP, Paterno D, Kastner S, Silverstein SM. Visual integration dysfunction in schizophrenia arises by the first psychotic episode and worsens with illness duration. J Abnorm Psychol. 2016;125(4):543–9. 27030995 10.1037/abn0000157 PMC4850085

[26] Dakin S, Frith U. Vagaries of visual perception in autism. Neuron. 2005;48(3):497–507. 16269366 10.1016/j.neuron.2005.10.018

[27] Pankow A, Katthagen T, Diner S, Deserno L, Boehme R, Kathmann N, Aberrant Salience Is Related to Dysfunctional Self-Referential Processing in Psychosis. Schizophr Bull. 2015;42(1):67–76. 26194892 10.1093/schbul/sbv098 PMC4681553

[28] Abraham A, Kaufmann C, Redlich R, Hermann A, Stark R, Stevens S, Self-referential and anxiety-relevant information processing in subclinical social anxiety: an fMRI study. Brain Imaging Behav. 2012;7(1):35–48. 10.1007/s11682-012-9188-x 22773051

[29] Spurr JM, Stopa L. Self-focused attention in social phobia and social anxiety. Clin Psychol Rev. 2002;22(7):947–75. 12238248 10.1016/s0272-7358(02)00107-1

[30] Mundy P, Gwaltney M, Henderson H. Self-referenced processing, neurodevelopment and joint attention in autism. Autism. 2010;14(5):408–29. 20926457 10.1177/1362361310366315 PMC2990352

[31] Itier RJ, Batty M. Neural bases of eye and gaze processing: The core of social cognition. Neurosci Biobehav Rev. 2009;33:843–63. 19428496 10.1016/j.neubiorev.2009.02.004 PMC3925117

[32] Nummenmaa L, Passamonti L, Rowe J, Engell AD, Calder AJ. Connectivity Analysis Reveals a Cortical Network for Eye Gaze Perception. Cereb Cortex. 2010;20:1780–7. 20016001 10.1093/cercor/bhp244 PMC2901016

[33] Georgescu AL, Kuzmanovic B, Schilbach L, Tepest R, Kulbida R, Bente G, Neural correlates of “social gaze” processing in high-functioning autism under systematic variation of gaze duration. NeuroImage Clin. 2013;3:340–51. 24273718 10.1016/j.nicl.2013.08.014 PMC3815020

[34] Pitskel NB, Bolling DZ, Hudac CM, Lantz SD, Minshew NJ, Vander Wyk BC, Brain Mechanisms for Processing Direct and Averted Gaze in Individuals with Autism. J Autism Dev Disord. 2011;41(12):1686–93. 21484518 10.1007/s10803-011-1197-x PMC3337548

[35] von dem Hagen EAH, Stoyanova RS, Rowe JB, Baron-Cohen S, Calder AJ. Direct Gaze Elicits Atypical Activation of the Theory-of-Mind Network in Autism Spectrum Conditions. Cereb Cortex. 2013;24(6):1485–92. 23324559 10.1093/cercor/bht003 PMC4014180

[36] Hasegawa N, Kitamura H, Murakami H, Kameyama S, Sasagawa M, Egawa J, Neural activity in the posterior superior temporal region during eye contact perception correlates with autistic traits. Neurosci Lett. 2013;549:45–50. 23792265 10.1016/j.neulet.2013.05.067

[37] Kohler CG, Loughead J, Ruparel K, Indersmitten T, Barrett FS, Gur RE, Brain activation during eye gaze discrimination in stable schizophrenia. Schizophr Res. 2008;99(1–3):286–93. 18248794 10.1016/j.schres.2007.09.038 PMC2276118

[38] Philip RCM, Dauvermann MR, Whalley HC, Baynham K, Lawrie SM, Stanfield AC. A systematic review and meta-analysis of the fMRI investigation of autism spectrum disorders. Neurosci Biobehav Rev. 2012;36(2):901–42. 22101112 10.1016/j.neubiorev.2011.10.008

[39] Kambeitz J, Kambeitz-Ilankovic L, Cabral C, Dwyer DB, Calhoun VD, van den Heuvel MP, Aberrant Functional Whole-Brain Network Architecture in Patients With Schizophrenia: A Meta-analysis. Schizophr Bull. 2016;42(suppl 1):S13–21. 27460615 10.1093/schbul/sbv174 PMC4960431

[40] Dong D, Wang Y, Chang X, Luo C, Yao D. Dysfunction of Large-Scale Brain Networks in Schizophrenia: A Meta-analysis of Resting-State Functional Connectivity. Schizophr Bull. 2017;44(1):168–81. 10.1093/schbul/sbx034 PMC5767956 28338943

[41] DeRosse P, Ikuta T, Karlsgodt KH, Peters BD, Gopin CB, Szeszko PR, White Matter Abnormalities Associated With Subsyndromal Psychotic-Like Symptoms Predict Later Social Competence in Children and Adolescents. Schizophr Bull. 2016;43(1):152–9. 27190281 10.1093/schbul/sbw062 PMC5216847

[42] Peters BD, Blaas J, de Haan L. Diffusion tensor imaging in the early phase of schizophrenia: What have we learned? J Psychiatr Res. 2010;44(15):993–1004. 20554292 10.1016/j.jpsychires.2010.05.003

[43] Jung M, Tu Y, Lang CA, Ortiz A, Park J, Jorgenson K, Decreased structural connectivity and resting-state brain activity in the lateral occipital cortex is associated with social communication deficits in boys with autism spectrum disorder. Neuroimage. 2019;190:205–12. 28927730 10.1016/j.neuroimage.2017.09.031

[44] Burrows CA, Laird AR, Uddin LQ. Functional connectivity of brain regions for self- and other-evaluation in children, adolescents and adults with autism. Dev Sci. 2016;19(4):564–80. 26750447 10.1111/desc.12400

[45] Friston KJ, Harrison L, Penny W. Dynamic causal modelling. Neuroimage. 2003;19(4):1273–302. 12948688 10.1016/s1053-8119(03)00202-7

[46] Mareschal I, Calder AJ, Dadds MR, Clifford CWG. Gaze categorization under uncertainty: psychophysics and modeling. J Vis. 2013;13(5):18. 10.1167/13.5.18 23608340

[47] Mareschal I, Otsuka Y, Clifford CWG. A generalized tendency toward direct gaze with uncertainty. J Vis. 2014;14(12):27. 10.1167/14.12.27 25342544

[48] Hecht H, Hörichs J, Sheldon S, Quint J, Bowers A. The effects of simulated vision impairments on the cone of gaze. Atten Percept Psychophys. 2015;77(7):2399–408. 26018645 10.3758/s13414-015-0931-4 PMC4607583

[49] Toh WL, Rossell SL, Castle DJ. Current visual scanpath research: a review of investigations into the psychotic, anxiety, and mood disorders. Compr Psychiatry. 2011;52(6):567–79. 21333977 10.1016/j.comppsych.2010.12.005

[50] Pelphrey K, Goldman BD, Pelphrey KA, Sasson NJ, Reznick JS, Paul G, Visual Scanning of Faces in Autism. J Autism Dev Disord. 2002;32(4):249–61. 12199131 10.1023/a:1016374617369

[51] Horley K, Williams LM, Gonsalvez C, Gordon E. Face to face: visual scanpath evidence for abnormal processing of facial expressions in social phobia. Psychiatry Res. 2004;127(1–2):43–53. 15261704 10.1016/j.psychres.2004.02.016

[52] Mundt JC, Marks IM, Shear MK, Greist JM. The Work and Social Adjustment Scale: a simple measure of impairment in functioning. Br J Psychiatry. 2002;180(5):461–4. 11983645 10.1192/bjp.180.5.461

[53] Peters E, Joseph S, Day S, Qarety P. Measuring Delusional Ideation: The 21-Item Peters et al. Delusions Inventory (PDI). Schizophrenia. 1998;(1994):1005–22. 10.1093/oxfordjournals.schbul.a007116 15954204

[54] Bell V, Halligan PW, Ellis HD. The Cardiff Anomalous Perceptions Scale (CAPS): A New Validated Measure of Anomalous Perceptual Experience. Schizophr Bull. 2005;32(2):366–77. 16237200 10.1093/schbul/sbj014 PMC2632213

[55] Andreasen N Scale for the Assessment of Negative Symptoms (SANS). Iowa City (US): University of Iowa; 1984.

[56] Baron-Cohen S, Wheelwright S, Skinner R, Martin J, Clubley E. The Autism Spectrum Quotient: Evidence from Asperger syndrome/high functioning autism, males and females, scientists and mathematicians. J Autism Dev Disord. 2001;31(1):5–17. 11439754 10.1023/a:1005653411471

[57] Hus V, Lord C. The Autism Diagnostic Observation Schedule, Module 4: Revised Algorithm and Standardized Severity Scores. J Autism Dev Disord. 2014;44(8):1996–2012. 24590409 10.1007/s10803-014-2080-3 PMC4104252

[58] Connor KM, Davidson JRT, Churchill LE, Sherwood A, Weisler RH, Foa E. Psychometric properties of the Social Phobia Inventory (SPIN). Br J Psychiatry. 2002;176(04):379–86. 10.1192/bjp.176.4.379 10827888

[59] Johnson HS, Inderbitzen-Nolan HM, Anderson ER. The Social Phobia Inventory: Validity and reliability in an adolescent community sample. Psychol Assess. 2006;18(3):269–77. 16953730 10.1037/1040-3590.18.3.269

[60] LeBeau RT, Glenn DE, Hanover LN, Beesdo-Baum K, Wittchen H-U, Craske MG. A dimensional approach to measuring anxiety for DSM-5. Int J Methods Psychiatr Res. 2012;21(4):258–72. 23148016 10.1002/mpr.1369 PMC6878356

[61] Wechlser D Wechsler Abbreviated Scale of Intelligence. 2nd ed. (WASI-II). San Antonio (TX, US): NCS Pearson; 2011.

[62] Gameroff MJ, Wickramaratne P, Weissman MM. Testing the Short and Screener versions of the Social Adjustment Scale-Self-report (SAS-SR). Int J Methods Psychiatr Res. 2012;21(1):52–65. 22139969 10.1002/mpr.358 PMC3433762

[63] Cornblatt BA, Auther AM, Niendam T, Smith CW, Zinberg J, Bearden CE, Preliminary findings for two new measures of social and role functioning in the prodromal phase of schizophrenia. Schizophr Bull. 2007;33(3):688–702. 17440198 10.1093/schbul/sbm029 PMC2526147

[64] Constantino JN, Gruber CP. Social Responsiveness Scale (SRS). Los Angeles (US): Western Psychological Services; 2005.

[65] Baron-Cohen S, Wheelwright S, Hill J, Raste Y, Plumb I. The Reading the Mind in the Eyes Test Revised Version: A Study with Normal Adults, and Adults with Asperger Syndrome or High-functioning Autism. J Child Psychol Psychiatry. 2001;42(2):241–51. 11280420

[66] Baron-Cohen S, Wheelwright S. The Empathy Quotient: An Investigation of Adults with Asperger Syndrome or High Functioning Autism, and Normal Sex Differences. J Autism Dev Disord. 2004;34(2):163–75. 15162935 10.1023/b:jadd.0000022607.19833.00

[67] McDonald S, Bornhofen C, Shum D, Long E, Saunders C, Neulinger K. Reliability and validity of The Awareness of Social Inference Test (TASIT): A clinical test of social perception. Disabil Rehabil. 2006;28(24):1529–42. 17178616 10.1080/09638280600646185

[68] Silverstein SM, Harms MP, Carter CS, Gold JM, Keane BP, MacDonald A III, Cortical contributions to impaired contour integration in schizophrenia. Neuropsychologia. 2015;75:469–80. 26160288 10.1016/j.neuropsychologia.2015.07.003 PMC4546547

[69] Langton SRH. The mutual influence of gaze and head orientation in the analysis of social attention direction. Q J Exp Psychol Sect A Hum Exp Psychol. 2000;53(3):825–45. 10.1080/713755908 10994231

[70] Palanica A, Itier RJ. Effects of Peripheral Eccentricity and Head Orientation on Gaze Discrimination. Vis Cogn. 2014;22(9–10):1216–32. 28344501 10.1080/13506285.2014.990545 PMC5362270

[71] Otsuka Y, Mareschal I, Calder AJ, Clifford CWG. Dual-route model of the effect of head orientation on perceived gaze direction. J Exp Psychol Hum Percept Perform. 2014;40(4):1425–39. 24730742 10.1037/a0036151 PMC4120707

[72] Lasagna CA, McLaughlin MM, Deng WY, Whiting EL, Tso IF. Deconstructing eye contact perception: Measuring perceptual precision and self-referential tendency using an online psychophysical eye contact detection task. PLoS One. 2020;15(3):1–20. 10.1371/journal.pone.0230258 PMC7069644 32168324

[73] Glasser MF, Sotiropoulos SN, Wilson JA, Coalson TS, Fischl B, Andersson JL, The minimal preprocessing pipelines for the Human Connectome Project. Neuroimage. 2013;80:105–24. 23668970 10.1016/j.neuroimage.2013.04.127 PMC3720813

[74] Behzadi Y, Restom K, Liau J, Liu TT. A component based noise correction method (CompCor) for BOLD and perfusion based fMRI. Neuroimage. 2007;37(1):90–101. 17560126 10.1016/j.neuroimage.2007.04.042 PMC2214855

[75] Roalf DR, Quarmley M, Elliott MA, Satterthwaite TD, Vandekar SN, Ruparel K, The impact of quality assurance assessment on diffusion tensor imaging outcomes in a large-scale population-based cohort. Neuroimage. 2016;125:903–19. 26520775 10.1016/j.neuroimage.2015.10.068 PMC4753778

[76] Stephan KE, Penny WD, Moran RJ, den Ouden HEM, Daunizeau J, Friston KJ. Ten simple rules for dynamic causal modeling. Neuroimage. 2010;49(4):3099–109. 19914382 10.1016/j.neuroimage.2009.11.015 PMC2825373

[77] Gordon EM, Laumann TO, Adeyemo B, Huckins JF, Kelley WM, Petersen SE. Generation and Evaluation of a Cortical Area Parcellation from Resting-State Correlations. Cereb Cortex. 2014;26(1):288–303. 25316338 10.1093/cercor/bhu239 PMC4677978

[78] Seitzman BA, Gratton C, Marek S, Raut RV, Dosenbach NUF, Schlaggar BL, A set of functionally-defined brain regions with improved representation of the subcortex and cerebellum. Neuroimage. 2020;206. 10.1016/j.neuroimage.2019.116290 PMC6981071 31634545

[79] Power J, Cohen A, Nelson S, Wig G, Barnes K, Church J, Functional Network Organization of the Human Brain. Neuron. 2011;72(4):665–78. 22099467 10.1016/j.neuron.2011.09.006 PMC3222858

[80] Behrens TEJ, Woolrich MW, Jenkinson M, Johansen-Berg H, Nunes RG, Clare S, Characterization and propagation of uncertainty in diffusion-weighted MR imaging. Magn Reson Med. 2003;50(5):1077–88. 14587019 10.1002/mrm.10609

[81] Behrens TEJ, Berg HJ, Jbabdi S, Rushworth MFS, Woolrich MW. Probabilistic diffusion tractography with multiple fibre orientations: What can we gain? Neuroimage. 2007;34(1):144–55. 17070705 10.1016/j.neuroimage.2006.09.018 PMC7116582

[82] Thakkar KN, van den Heiligenberg FMZ, Kahn RS, Neggers SFW. Speed of saccade execution and inhibition associated with fractional anisotropy in distinct fronto-frontal and fronto-striatal white matter pathways. Hum Brain Mapp. 2016;37(8):2811–22. 27091670 10.1002/hbm.23209 PMC6867450

[83] Yao B, Neggers SFW, Rolfs M, Rösler L, Thompson IA, Hopman HJ, Structural Thalamofrontal Hypoconnectivity Is Related to Oculomotor Corollary Discharge Dysfunction in Schizophrenia. J Neurosci. 2019;39(11):2102–13. 30630882 10.1523/JNEUROSCI.1473-18.2019 PMC6507081

[84] Alexander AL, Hurley SA, Samsonov AA, Adluru N, Hosseinbor AP, Mossahebi P, Characterization of Cerebral White Matter Properties Using Quantitative Magnetic Resonance Imaging Stains. Brain Connect. 2011;1(6):423–46. 22432902 10.1089/brain.2011.0071 PMC3360545

[85] Sripada C, Angstadt M, Rutherford S, Kessler D, Kim Y, Yee M, Basic Units of Inter-Individual Variation in Resting State Connectomes. Sci Rep. 2019 Feb 13;9(1):1900. 30760808 10.1038/s41598-018-38406-5 PMC6374507

[86] Sripada C, Rutherford S, Angstadt M, Luciana M, Thompson WK, Weigard A, Prediction of Neurocognitive Profiles in Youth From Resting State fMRI. Mol Psychiatry. 2019. doi: 10.1038/s41380-019-0481-6 PMC7055722 31427753

[87] Balsters JH, Mantini D, Apps MAJ, Eickhoff SB, Wenderoth N. Connectivity-based parcellation increases network detection sensitivity in resting state {fMRI}: An investigation into the cingulate cortex in autism. NeuroImage Clin. 2016;11:494–507. 27114898 10.1016/j.nicl.2016.03.016 PMC4832089

[88] Bohlken MM, Brouwer RM, Mandl RCW, Van den Heuvel MP, Hedman AM, De Hert M, Structural Brain Connectivity as a Genetic Marker for Schizophrenia. JAMA Psychiatry. 2016;73(1):11. 26606729 10.1001/jamapsychiatry.2015.1925

[89] Hassel S, Almeida JRC, Kerr N, Nau S, Ladouceur CD, Fissell K, Elevated striatal and decreased dorsolateral prefrontal cortical activity in response to emotional stimuli in euthymic bipolar disorder: no associations with psychotropic medication load. Bipolar Disord. 2008;10(8):916–27. 19594507 10.1111/j.1399-5618.2008.00641.x PMC2711546

[90] Woods SW. Chlorpromazine Equivalent Doses for the Newer Atypical Antipsychotics. J Clin Psychiatry. 2003;64(6):663–7. 12823080 10.4088/jcp.v64n0607

